# Fast Switching and High Polarization in Ferroelectric Hf_0.5_Zr_0.5_O_2_ Films

**DOI:** 10.1002/smsc.202500465

**Published:** 2026-01-15

**Authors:** Faizan Ali, Tingfeng Song, Florencio Sánchez, Ignasi Fina

**Affiliations:** ^1^ Institut de Ciència de Materials de Barcelona (ICMAB‐CSIC) Campus UAB 08193 Bellaterra Spain; ^2^ Department of Applied Physics The Hong Kong Polytechnic University Hong Kong China; ^3^ Joint Research Center of Microelectronics The Hong Kong Polytechnic University Hong Kong China

**Keywords:** defect dipoles, domain dynamics, domain wall, ferroelectric HfO_2_, polarization switching kinetics

## Abstract

HfO_2_‐based ferroelectric thin films exhibit promising potential for next‐generation nonvolatile memories and neuromorphic devices. Achieving both high polarization and fast switching is required, but optimizing one of them can be at the cost of degrading the other. This study achieves simultaneous fast switching and high polarization in Hf_0.5_Zr_0.5_O_2_ epitaxial films grown by pulsed laser deposition. The influence of redox conditions during film growth on the ferroelectric switching kinetics and domain wall motion is systematically explored. Switching spectroscopy and Rayleigh analysis reveal that optimized redox conditions, tuned by oxygen and argon pressures during Hf_0.5_Zr_0.5_O_2_ deposition, enable both enhanced polarization and switching speeds. Switching time can be further shortened when the final polarization state is aligned with the internal electric fields. The study challenges the trade‐off between switching speed and polarization, demonstrating that precise control of defects in the film can simultaneously optimize both.

## Graphical abstract

Ferroelectric Hf_0.5_Zr_0.5_O_2_ thin films are grown under tuned redox conditions to simultaneously achieve high polarization and fast switching. Control of defect dipoles and internal fields enables stable domain wall motion without compromising performance. This work provides a pathway for designing fast and energy‐efficient ferroelectric devices.

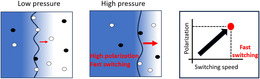

## 1 Introduction

Ferroelectric materials have gained significant attention due to their unique polarization switching behavior, which is particularly important for applications in energy‐efficient neuromorphic computing and nonvolatile memories.^[^ ^1^, ^2^, ^3^, ^4^, ^5^, ^6^ ^]^ Among them, ferroelectric HfO_2_‐based materials have emerged as the most promising candidates due to their compatibility with complementary metal‐oxide‐semiconductor processes and robust ferroelectricity at ultra‐small thicknesses.^[^ ^7^, ^8^, ^9^ ^]^ The ferroelectric properties of HfO_2_‐based materials are extremely sensitive to deposition conditions, including temperature and ambient gas composition and pressure, which influence the phases that crystallize and defects formation.^[^ ^10^, ^11^, ^12^, ^13^ ^]^ In particular, redox conditions during synthesis play a crucial role in modulating oxygen availability, which in turn affects the amount of oxygen vacancies and the phases that crystallize. Crystal phases, but also oxygen vacancies, impact key switching characteristics of HfO_2_‐based films, such as polarization, coercive field, and switching speed.^[^ ^14^, ^15^ ^]^ For instance, it has been shown that Hf_0.5_Zr_0.5_O_2_ polycrystalline films deposited at low oxygen doses exhibit faster switching but at the cost of reduced polarization.^[^ ^15^ ^]^ Similarly, oxygen‐deficient conditions have been reported to favor the stabilization of the ferroelectric orthorhombic or tetragonal phases over the nonpolar monoclinic phase, while oxygen‐rich conditions promote monoclinic growth, resulting in suppressed ferroelectricity in HfO_2_ polycrystalline films.^[^ ^16^ ^]^

In addition to deposition conditions, doping concentration is critical to stabilize the ferroelectric phase and tailor switching dynamics.^[^ ^3^, ^17^ ^]^ For example, Ga‐doped HfO_2_ polycrystalline films with optimal Ga concentration have been shown to simultaneously enhance polarization and enable faster switching, possibly due to variation of the switching mechanism.^[^ ^18^ ^]^ On the other hand, in Hf_1‐*x*_Zr_*x*_O_2_, increasing the Zr content toward *x* = 0.5 (i.e., Hf_0.5_Zr_0.5_O_2_) maximizes polarization but results in slower switching speeds.^[^ ^19^ ^]^ Additionally, film architecture, such as thickness and vertical composition gradients, further modulates switching behavior. Ultrathin films (<10 nm) typically show enhanced polarization due to size effects, but their switching dynamics are typically slower compared to thicker films (e.g., 10–20 nm).^[^ ^20^, ^21^, ^22^, ^23^ ^]^ This inverse correlation arises from smaller grain sizes, increased grain boundary density, and asymmetric interfacial layers in orthorhombic phase‐rich thin films, which introduce domain pinning and broaden the activation field distribution.^[^ ^22^, ^23^ ^]^ Moreover, compositionally graded films, with a vertical gradient in Hf/Zr ratio, have recently demonstrated a compelling combination of high polarization and fast switching compared to single‐layer films, a positive effect attributed to the integration of phase and domain engineering.^[^ ^24^ ^]^

Understanding the intrinsic switching dynamics of hafnia‐based ferroelectrics is crucial for optimizing both polarization and reliability in device applications. However, the interplay between polarization and switching speed remains complex, with conflicting trends reported in the literature, as aforementioned, reflecting the delicate balance between defect chemistry, phase stability, and domain wall kinetics. These contradictions highlight the complexity of switching dynamics. In this context, epitaxial films, which possess more controlled microstructures compared to polycrystalline films, serve as model systems for probing intrinsic switching behavior and understanding performance metrics such as polarization, piezoelectric response, endurance, and retention.^[^ ^25^, ^26^, ^27^, ^28^, ^29^, ^30^ ^]^ Ferroelectric properties have been demonstrated in sub‐5 nm epitaxial Hf_0.5_Zr_0.5_O_2_ films grown on SrTiO_3_(001) substrates by pulsed laser deposition (PLD), with oxygen pressure during deposition playing a key role in modulating their structural and ferroelectric characteristics.^[^ ^28^ ^]^ In particular, the films deposited at high oxygen pressure exhibited high polarization at the expense of severe fatigue, and vice versa for low oxygen pressure.^[^ ^31^ ^]^

While low oxidation conditions have been shown to enhance polarization in HfO_2_‐based films deposited by sputtering and atomic layer deposition, which is consistent with theoretical predictions linking oxygen vacancies to phase stabilization.^[^ ^13^, ^16^, ^32^, ^33^, ^34^ ^]^ However, PLD‐grown films usually exhibit the opposite trend.^[^ ^28^ ^]^ This is a consequence of the fact that the high plasma energy in PLD strongly affects film growth under low‐pressure oxygen‐deficient conditions. Indeed, the energy of ablated species that propagate under vacuum can be even hundreds of eV, and they can cause self‐sputtering when impinging on the film.^[^ ^35^ ^]^ Increasing oxygen pressure reduces plasma energy, but this reduces the number of oxygen vacancies in the film. Thus, achieving optimal ferroelectric properties requires carefully balancing oxidation conditions and plasma energy. To overcome this limitation, a mixture of reactive oxygen and inner argon can be introduced in the PLD chamber.^[^ ^36^ ^]^ The partial pressure of oxygen determines the number of oxygen vacancies, while argon is added to increase the total pressure. This permits the optimization of the energy of the ablated species, since a moderately high energy is positive considering roughness^[^ ^37^, ^38^ ^]^ and crystallinity^[^ ^39^ ^]^ of the films. Moreover, if pressure is too high, the ablated species would be scattered, causing off‐stoichiometry by preferential loss of light elements.^[^ ^40^, ^41^ ^]^ Considering the relevance of oxygen vacancies on the stabilization of the ferroelectric phase of HfO_2_, the combination of argon and oxygen appears to be convenient to grow ferroelectric HfO_2_ films by PLD. We demonstrated that this strategy permits growing Hf_0.5_Zr_0.5_O_2_ epitaxial films under optimized low oxidation conditions, resulting in higher content of orthorhombic phase and higher polarization.^[^ ^42^ ^]^ In addition, this approach not only enhances ferroelectric polarization but also preserves endurance and retention.^[^ ^43^ ^]^ However, the influence on switching speed is unknown.

In this work, we systematically investigate the effect of the Ar/O_2_ atmosphere during PLD on the switching dynamics of epitaxial Hf_0.5_Zr_0.5_O_2_ films. We show that the combined tuning of oxidation conditions and plasma energy, enabled through precise control of Ar and O_2_ partial pressures, creates favorable growth conditions for achieving both high polarization and ultrafast switching. These results highlight a robust strategy for addressing the long‐standing polarization–switching speed trade‐off in ferroelectric HfO_2_ systems.

## 2 Results and Discussion

**Figure 1:**
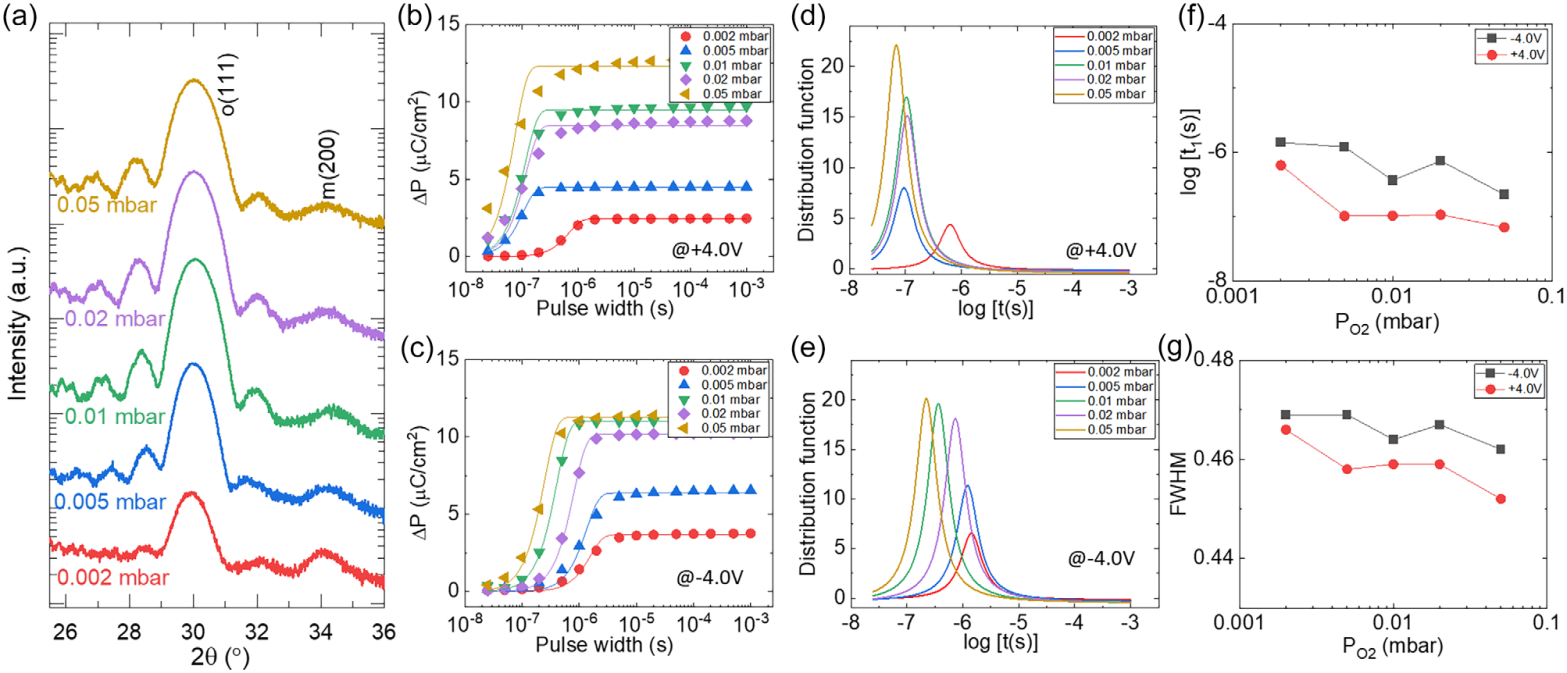
a) XRD *θ*–2*θ* scans of the series of films deposited under an Ar pressure of 0.1 mbar and varying oxygen pressures (0.002–0.05 mbar). Δ*P* as a function of pulse width under b) *a* + 4.0 V writing pulse and c) *a* − 4.0 V writing pulse. Solid lines represent fits using the NLS model. d,e) Corresponding Lorentzian distribution functions fitted to the switching data for positive and negative polarities, respectively. f) Extracted switching time (log[*t*_1_(*s*)]) and g) FWHM of the distribution as a function of oxygen pressure.

**Figure** **1**a shows the X‐ray diffraction (XRD) *θ*–2*θ* scans of the series of films grown under *P*_Ar_ (0.1 mbar), while varying *P*_O2_ from 0.002 to 0.05 mbar. The peak at about 30°, accompanied by Laue fringes, corresponds to the (111) reflection of the ferroelectric orthorhombic phase. The very low intensity peak at about 34.5° corresponds to the (002) reflection of the nonferroelectric monoclinic phase. More detailed XRD characterization is reported elsewhere.^[^ ^42^, ^43^ ^]^ To further assess the effect of redox conditions on the defect landscape, X‐ray photoelectron spectroscopy (XPS) measurements were performed for films grown under varying oxygen pressures at a fixed argon atmosphere of 0.1 mbar. The full XPS spectra of all films can be found in Figure S1, Supporting Information. In the Hf 4*f* spectra of the spin‐orbit doublet of the Hf 4*f*_7/2_ peak centered at a binding energy of 17.58 eV (Figure S2, Supporting Information), no variation among samples is observed. In fact, presence of reduced Hf, discernible by observation of sub‐oxide shoulders, variations of it, or binding‐energy shifts variations are not observed indication that samples are stochiometric with variations of the amount of the oxygen vacancies below the detectivity of the technique^[^ ^44^, ^45^, ^46^ ^]^ (Figure S1 and S2, Supporting Information) indicating that the oxygen vacancy concentration and variations of oxygen vacancy concentrations among samples are below the detection limit. Although the direct detection of oxygen vacancy variation by XPS was not conclusive, the photo‐response measurements can provide indirect evidence regarding defect concentration or the presence of bad interfaces.^[^ ^47^, ^48^, ^49^ ^]^ Polarization–voltage (*P*–*V*) hysteresis loops measured under laser illumination and in the dark (Figure S3, Supporting Information) exhibit virtually identical remanent polarization and coercive voltage values. The absence of any light‐induced modification in switching behavior confirms that photoexcited carriers or defect‐related trap states do not contribute appreciably to the polarization response. Additionally, as shown in Figure S4, Supporting Information, the breakdown voltage increases systematically with increasing oxygen pressure, indicating that the density of oxygen‐vacancy defects decreases under more oxidizing growth conditions. In fact, as shown in Figure S5, Supporting Information, a clear relation between breakdown electric voltage and switching time has been observed, indicating a link between oxygen vacancies and switching time. Therefore, it can be concluded that the amount of oxygen vacancies in the films is small, and that any differences in their concentration among the films are present but not detectable by commonly used techniques.

The switching dynamics of epitaxial Hf_0.5_Zr_0.5_O_2_ films are summarized in Figure 1b,c, which show the dependence of Δ*P* on switching pulse duration for positive and negative writing biases, respectively. Data reveal substantial variation in switching kinetics response depending on *P*_O2_. Δ*P* values are extracted following the subtraction of residual leakage current contributions, which are characteristics of ultra‐thin ferroelectric films.^[^ ^50^, ^51^ ^]^ The correction method follows our earlier work,^[^ ^30^ ^]^ and further details are provided in the Supporting Information Figure S6. The raw polarization data, prior to leakage current subtraction, are shown in Figure S7, Supporting Information. Figure 1b,c shows that all films exhibit a steep rise in polarization over time, indicating ferroelectric switching.

The polarization reversal kinetics in ferroelectric materials are governed by the interplay between domain nucleation and domain wall motion. For hafnia‐based systems, it has been reported that domain growth is restricted by inhomogeneous nucleation. In this scenario, the time‐dependent switched polarization is well‐described by the nucleation‐limited‐switching (NLS) model.^[^ ^52^ ^]^

(1) $$\Delta P=2P_{r}\int_{-\infty }^{+\infty }\left[1-e^{-{\left(\frac{t}{\tau }\right)}^{n}}\right]F\left(\log\tau \right)\times d(\log\tau )$$

where *n* is the effective dimension (we fix it to 2, as for thin films expansion along the out‐of‐plane direction is expected not to contribute significantly), *t* is the pulse width in our experiments, *τ* is the characteristic switching time, and *F*(log*τ*) is the Lorentzian distribution for the logarithm of switching time. The Lorentzian distribution can be expressed as.

(2) $$F\left(\text{log}\tau \right)=\frac{A}{\pi }\left[\frac{w}{{\left(\text{log}\tau -\text{log}t_{1}\right)}^{2}+w^{2}}\right]$$

where *A* is a normalization constant, *w* is the full width at half maxima (FWHM), and log *t*_1_ is the central value of the Lorentzian distribution for the logarithm of switching time. Fittings using the NLS are included in Figure 1b,c.

To gain deeper insight into switching kinetics, Lorentzian distributions are shown in Figure 1d,e for positive and negative writing biases, respectively. The peaks progressively shift toward shorter switching times as *P*_O2_ increases, indicating faster domain reversal. Typically, the two main characteristics of a Lorentzian peak, namely log [*t*_1_(*s*)] and FWHM, offer intuitive insights into the switching dynamics. While log [*t*_1_(*s*)] reflects the average switching speed, FWHM is indicative of the spread of the distribution of switching nucleation events. Figure 1f,g summarizes the extracted log [*t*_1_(*s*)] and FWHM as a function of *P*_O2_, respectively.

Under positive reading (negative writing) bias, a clear nonmonotonic trend is observed. As *P*_O2_ increases from 0.002 to 0.05 mbar, the switching time progressively decreases, reaching a minimum at 0.05 mbar (log [*t*_1_(*s*)] ≈ −6.65), i.e., 223 ns, indicative of the fastest switching condition (Figure 1f). This enhanced switching performance is likely due to the minimal concentration of oxygen vacancies promoting the stabilization of the orthorhombic phase. FWHM (Figure 1g) decreases with pressure as a general trend, implying a more uniform switching nucleation, again likely stemming from a lower content of oxygen vacancies. Thus, switching dynamics under positive reading bias are governed by a delicate balance between oxygen vacancy concentration and the structural quality of the films, with the former dominating at low to intermediate *P*_O2_ and the latter playing a critical role at higher oxygen pressures.

Interestingly, the switching behavior under negative reading polarity follows a similar but more pronounced trend (Figure 1f). The switching time steadily decreases with increasing *P*_O2_, reaching 67 ns (log [*t*_1_(*s*)] ≈ −7.17) at 0.05 mbar, which is even faster than the best positive reading polarity condition. This asymmetry between positive and negative polarities across all oxygen pressures points to the presence of internal built‐in fields or asymmetric defect distributions.^[^ ^53^ ^]^ Such asymmetry can arise from electrode‐induced differences in oxygen vacancy accumulation, interface quality, or the presence of interfacial dead layers.^[^ ^53^, ^54^, ^55^, ^56^ ^]^ The low signal of reduced hafnia observed in the XPS characterizations (Figure S2, Supporting Information) does not allow us to unambiguously identify the underlying mechanism. Overall, faster switching is observed when a positive voltage is applied to the top electrode during the writing pulse. This indicates the presence of a small internal (imprint) field that assists switching under positive bias. Such behavior suggests that the built‐in field most likely originates from the work‐function difference between the Pt top (5.7 eV)^[^ ^57^ ^]^ and the La_0.67_Sr_0.33_MnO_3_ (LSMO) LSMO bottom (4.9 eV)^[^ ^58^ ^]^ electrodes. Other effects, such as chemistry at the electrode/ferroelectric interface^[^ ^59^, ^60^, ^61^, ^62^ ^]^ or strain gradients,^[^ ^63^ ^]^ can be at the origin of the observed imprint.

**Figure 2:**
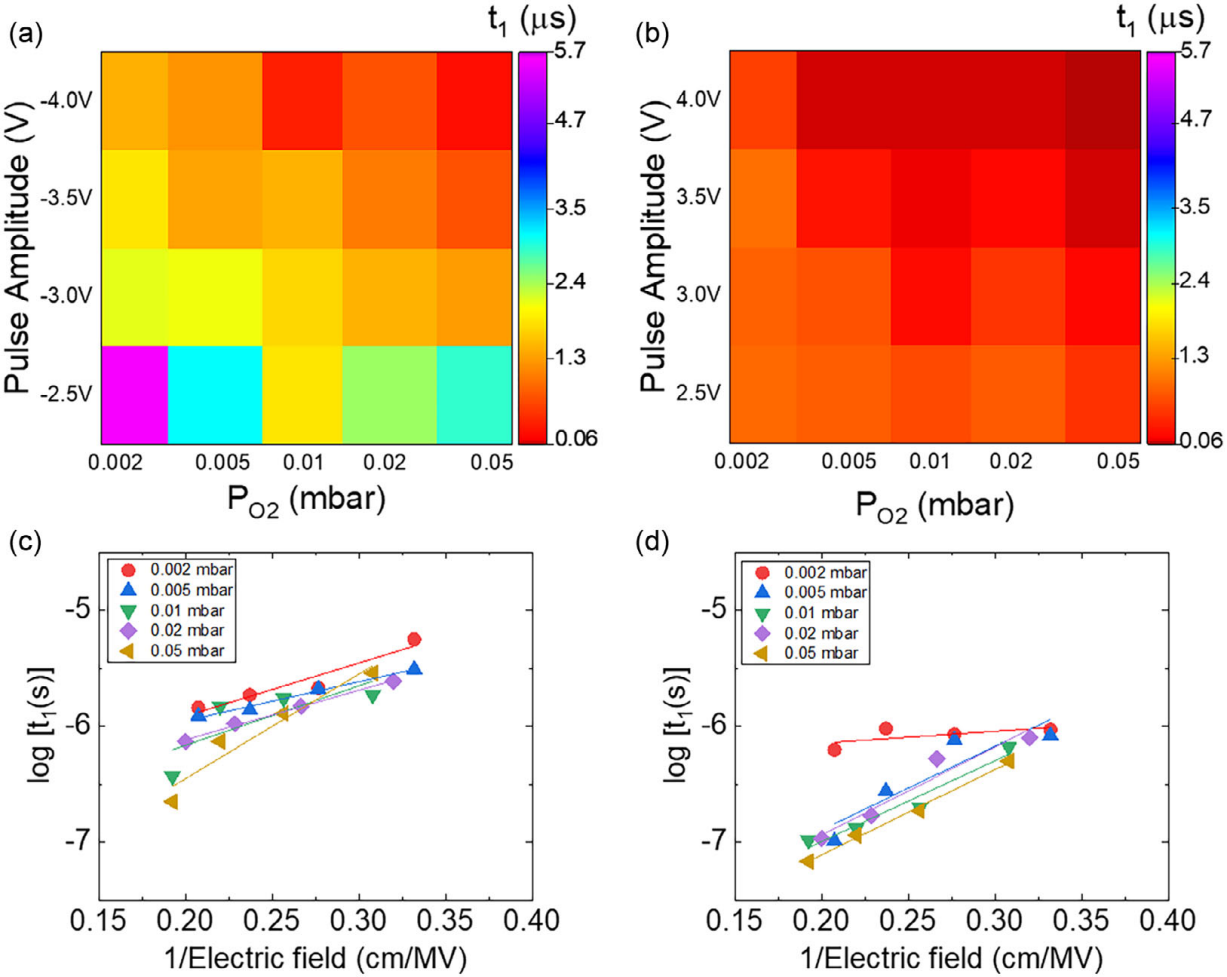
Switching time (*t*_1_) maps of films as a function of oxygen pressure and pulse amplitude under a) negative and b) positive writing bias. c,d) Relationship between log[*t*_1_(*s*)] and the inverse electric field for negative and positive writing polarities, respectively. The data in (c,d) are fitted using Merz's law.

**Figure** **2** illustrates the field‐dependent switching dynamics of films grown under varying oxygen pressure, providing deeper insights into the role of the applied voltage and defect landscape on domain reversal kinetics. The switching time is presented as a function of *P*_O2_ and pulse amplitude for both negative (Figure 2a) and positive writing (Figure 2b) polarities. In the negative polarity regime, the switching time generally decreased (faster switching) with increasing pulse amplitude for all *P*_O2_ conditions. Notably, this *P*_O2_‐dependent switching behavior is more pronounced at low voltages (e.g., −2.5 V). Specifically, the switching time was 5.6 μs for 0.002 mbar films, whereas it was only 2.9 μs for 0.05 mbar films, further demonstrating the most homogeneous switching in samples grown at larger *P*_O2_. In the positive‐polarity regime, a more consistent trend was observed. The switching time monotonically decreased with increasing pulse amplitude across all *P*_O2_ conditions. Moreover, the relationship between the switching time and the inverse of the electric field follows Merz's law^[^ ^64^ ^]^ for both positive (Figure 2c) and negative (Figure 2d) polarities.

**Figure 3:**
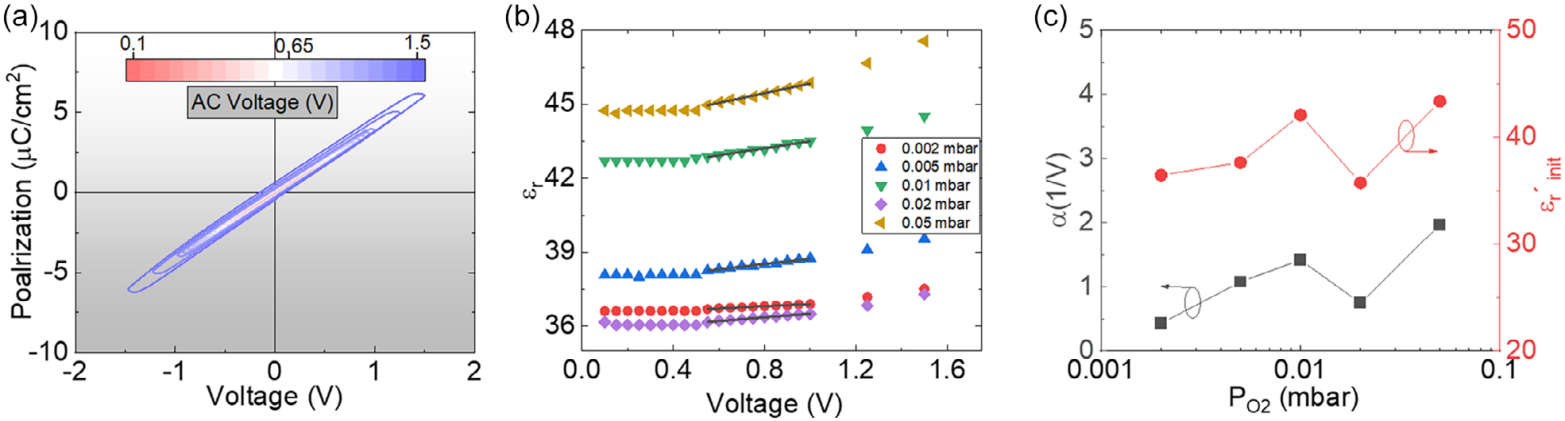
a) Polarization–voltage (*P*–*V*) dependency of a representative film deposited at *P*_Ar_ = 0.1 mbar and *P*_O2_ = 0.05 mbar for the indicated *V*_AC_, showing a weak response at voltages lower than *V*_c_. b) Voltage dependence of the dielectric permittivity (*ε*_r_) for films deposited under constant *P*_Ar_ (0.1 mbar) with varying *P*_O2_ (0.002–0.05 mbar). c) Dependence of the Rayleigh parameters, initial permittivity (*ε*′_r init_), and Rayleigh coefficient (*α*), on oxygen pressure. The solid black lines in Figure 3b exhibit curve fitting using the Rayleigh equation.

To further elucidate the contribution of domain wall motion to the ferroelectric response of Hf_0.5_Zr_0.5_O_2_ thin films, Rayleigh analysis was performed. **Figure** **3**a displays the polarization–AC voltage (*P*–*V*_AC_) hysteresis loops of the film deposited at 0.1 mbar *P*_Ar_ and 0.05 mbar *P*_O2_. The *P*–*V*_AC_ loops remain nearly linear for all measured voltages, which are below the coercive voltage (*V*_c_), confirming that no irreversible domain switching occurs in this range. The small aperture is mainly contributed to by a series resistance effect. Figure 3b shows the dielectric permittivity as a function of excitation AC voltage for films grown under varying *P*_O2_ and constant *P*_Ar_ (0.1 mbar), extracted from data equivalent to that shown in Figure 3a for all the samples. The extracted dielectric permittivity (*ε*_r_) values range from ≈36 to 48. These values are slightly higher than our earlier findings,^[^ ^65^ ^]^ which may be attributed to optimized Ar/O_2_ growth conditions improving structural quality and reducing defect‐related contributions. In addition, the values of *ε*_r_ were calculated using polarization loops measured at low voltages (<*V*_c_), where the motion or displacement of the pre‐existing domain walls contributes. The slope of the polarization curve corresponds to the *ε*_r_. The relationship is given as

(3) $$P=\varepsilon_{0}\varepsilon_{r}V$$

where *ε*_0_ is the vacuum permittivity, and *V* is the applied voltage.

According to Figure 3b, at low voltages (0.1–0.5 V), which lie below the characteristic threshold voltage (*V*_th_), the films exhibit behavior consistent with the so‐called subthreshold regime. In this region, no irreversible domain wall motion occurs.^[^ ^66^ ^]^ Instead, domain walls undergo fast reversible oscillations within local potential wells formed by defect pinning sites.^[^ ^67^ ^]^ Such a reversible domain wall response, combined with intrinsic domain wall propagation displacements, gives rise to a voltage amplitude‐independent polarization response. However, when the voltage exceeds the threshold value of 0.55 V, large‐scale irreversible domain wall motion is initiated across the defect landscape, resulting in noticeable nonlinearity in the polarization response. The dielectric permittivity–AC voltage (*ε*′_r_–*V*_AC_) characteristics of Hf_0.5_Zr_0.5_O_2_ films deposited under different oxygen pressures show a linear dependence within the voltage range of 0.55–1 V (Figure 3b), consistent with typical Rayleigh‐like behavior. This range is therefore defined as the Rayleigh region for our analysis. The dielectric response in this region can be described by the Rayleigh equation.^[^ ^68^ ^]^

(4) $$\varepsilon_{r}^{\prime }\left(V\right)=\varepsilon_{\text{r init}}^{\prime }+\text{α}V$$

where *ε*′_r init_ is the initial dielectric permittivity at zero voltage and *α*V represents the contribution of irreversible domain wall motion to *ε*′_r_.^[^ ^69^ ^]^ Figure 3c shows the Rayleigh parameters as a function of *P*_O2_. Both parameters exhibit a clear increasing trend, echoing the switching kinetics discussed earlier, except for the films deposited at 0.02 mbar *P*_O2_. The Rayleigh coefficient *α*, which quantifies domain wall contributions, thus their mobility, increases with *P*_O2_ from 0.002 to 0.05 mbar, reaching 1.97 V^−1^ for the film grown under 0.5 mbar of *P*_O2_. This increase is likely due to improved structural homogeneity and phase purity at higher oxygen pressure, which reduces defect‐related pinning and facilitates more coherent domain wall propagation. For the sample grown under 0.02 mbar of *P*_O2_, *α* decreases to 0.75 V^−1^, reflecting reduced domain wall activity, likely due to local structural inhomogeneities and a corresponding increase in pinning effects of this particular sample. Similarly, *ε*′_r init_ also follows the same trend as *α*, increasing from 36.5 at 0 mbar *P*_O2_ to 43.4 at 0.05 mbar *P*_O2_, with a local minimum at 0.02 mbar *P*_O2_ (35.7). The increase in *ε*′_r init_ indicates enhanced dielectric responsiveness and lower energy barriers for domain motion under higher *P*_O2_.

**Figure 4:**
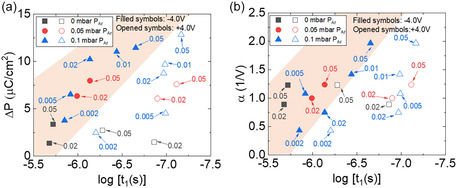
a) Switched polarization (Δ*P*) and switching time (log[*t*_1_(*s*)]) of films as a function of oxygen pressure for three different argon partial pressures (0, 0.05, and 0.1 mbar), under both positive (empty symbols) and negative (filled symbols) writing bias. b) Correlation between the Rayleigh coefficient (*α*) and switching time (log[*t*_1_(*s*)]) for the same set of samples.

The switching dynamics of Hf_0.5_Zr_0.5_O_2_ films are strongly influenced by the background gas composition during deposition, particularly the relative concentrations of oxygen and argon. **Figure** **4**a presents the extracted switching time and switched polarization of the films deposited at varying *P*_O2_ and under three fixed Ar partial pressures: 0, 0.05, and 0.1 mbar for both positive and negative writing polarities. The raw and corrected data of 0 mbar *P*_Ar_ and 0.05 mbar *P*_Ar_ series can be found in (Figure S8 and S9, Supporting Information). In general, the switching time decreases, and the polarization increases with increasing *P*_Ar_ and *P*_O2_. The 0 mbar *P*_Ar_ series exhibits relatively long switching times (1.9 μs, log [*t*_1_(*s*)] ≈ −5.72) and reduced Δ*P* (3.4 μC cm^2^ for negative writing polarity), particularly for *P*_O2_ = 0.05 mbar. In contrast, the 0.05 mbar *P*_Ar_ series shows improved performance. For instance, the films deposited at 0.05 mbar *P*_Ar_ and 0.05 mbar *P*_O2_ exhibit a shorter switching time of 0.7 μs (log [*t*_1_(*s*)] ≈ −6.13) and enhanced polarization (8.0 μC cm^2^). Moreover, in the case of 0.1 mbar *P*_Ar_, the switching time and switched polarization are further improved. For example, the films deposited at 0.1 mbar *P*_Ar_ and 0.05 mbar *P*_O2_ exhibit fast switching (223 ns, log [*t*_1_(*s*)] ≈ −6.65) and high polarization (11.5 μC cm^2^). Interestingly, the results challenge the switching–polarization dilemma, wherein faster switching leads to reduced polarization during incomplete domain reversal.^[^ ^15^, ^19^, ^20^, ^30^ ^]^ In our case, faster switching correlates with higher Δ*P*, especially in the 0.1 Ar series at 0.05 mbar *P*_O2_, where optimized oxygen vacancy concentrations and reduced defect pinning allow for both rapid and complete ferroelectric switching. This synergy suggests that under optimized growth conditions, the trade‐off between switching speed and switchable polarization can be mitigated. Additionally, the effect of Ar becomes increasingly pronounced at higher oxygen pressure. At *P*_O2_ = 0.02 mbar, increasing *P*_Ar_ from 0 to 0.1 mbar results in only a modest enhancement in switching time (improving from 2.0 μs, log [*t*_1_(*s*)] = −5.68 to 0.7 μs, log [*t*_1_(*s*)] = −6.13), indicating that it is still limited by the presence of oxygen vacancies and other structural defects (Figure S10a, Supporting Information). In contrast, at *P*_O2_ = 0.05 mbar, the same Ar increase yields a more substantial improvement in switching kinetics time (from 1.9 μs, log [*t*_1_(*s*)] = −5.72 to 223 ns, log [*t*_1_(*s*)] = −6.65) (Figure S10b, Supporting Information). This suggests that under sufficiently oxidizing conditions, Ar effectively reduces defect density by moderating plasma energy, thereby minimizing nonoxygen‐vacancy defects and improving phase uniformity. This highlights the interaction between oxygen and argon in tailoring the defect landscape and enhancing ferroelectric performance. Similar trends were also observed for positive writing polarity (empty symbols), but with faster switching time due to the influence of the internal built‐in field that assists in domain reversal under positive bias, reaching values as low as 67 ns for the *P*_Ar_ = 0.1 mbar sample.

Figure 4b further examines the relationship between switching dynamics and domain wall mobility by plotting *α* against the log [*t*_1_(*s*)] for the same set of samples. This comparative analysis provides deeper insight into how the interplay of oxygen and argon pressures governs irreversible domain wall motion. The 0.1 mbar *P*_Ar_ series again shows the most significant evolution, with *α* increasing as switching time decreases, confirming that faster switching correlates with enhanced irreversible domain wall activity. For example, film deposited with 0.1 mbar Ar and 0.05 mbar O_2_ exhibits the shortest switching time of 67 ns (log [*t*_1_(*s*)] ≈ −7.17) and the highest *α* value (1.97 V^−1^), highlighting optimal conditions for both kinetics and polarization. In contrast, the 0 and 0.05 mbar Ar series show a narrower range of variation in both *α* and log [*t*_1_(*s*)]. Notably, at *P*_O2_ = 0.02 mbar, the impact of increasing *P*_Ar_ is relatively minor, with *α* values remaining modest (0.75–1.00 V^−1^) and switching times improving only slightly. However, at *P*_O2_ = 0.05 mbar, the combined effect of higher oxygen and argon pressures becomes more pronounced, leading to stronger domain wall contributions (*α* = 1.23–1.97 V^−1^) and improved switching behavior. Together, Figure 4a,b reinforce the notion that, under carefully optimized oxygen and argon pressures, both rapid switching and strong irreversible domain wall motion can be achieved.

**Figure 5:**
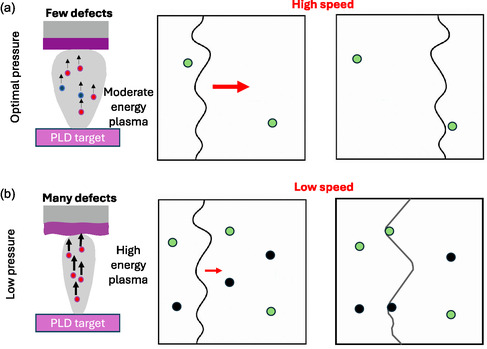
Schematic illustration of the growth of ferroelectric HfO_2_ films under different atmosphere conditions, the resulting defects formed, and the impact on domain wall motion. a) In Ar‐assisted PLD with optimized argon and oxygen partial pressures, scattering by the background gas reduces the kinetic energy of ablated species, leading to smoother film growth with fewer defects. b) In conventional PLD under low‐pressure oxygen, the ablated species experience less scattering and thus possess higher kinetic energy (indicated by larger black arrows), which enhances defect formation, such as oxygen vacancies and other structural imperfections. These defects act as pinning centers that impede domain wall motion and slow ferroelectric switching. In the schematics, black arrows represent the motion and relative kinetic energy of ablated particles during deposition, red arrows indicate the relative speed and direction of domain wall motion, red and blue circles denote oxygen and argon atoms, respectively, green circles represent oxygen vacancies, and black circles denote other defects.

**Figure** **5** schematically illustrates the impact of defect concentration on domain wall mobility. The film growth by optimal atmosphere conditions with a moderately low pressure of oxygen (to favor orthorhombic phase formation and thus increase the polarization) and added argon gas to reduce the energy of the PLD plasma is schematically shown in Figure 5a. In these films, the lower density of charged defects leads to reduced pinning, enabling smoother and faster domain wall motion, reflected in higher *α* and faster switching dynamics. The growth under low pressure of pure oxygen is illustrated in Figure 5b. In these films, the accumulation of defects can introduce local pinning that hinders domain wall propagation, resulting in slower switching dynamics and reduced Rayleigh coefficients. This scenario correlates with the slower switching time and reduced *α* values observed for films grown at low oxygen pressure or without argon gas, where higher defect densities (oxygen vacancies or others) dominate.

Supporting Information Table S1 summarizes the remanent polarization and switching time of our epitaxial Hf_0.5_Zr_0.5_O_2_ films in comparison with representative literature data.^[^ ^21^, ^70^, ^71^, ^72^, ^73^, ^74^, ^75^ ^]^ The optimized film grown at *P*_O2_ = 0.05 mbar exhibits a high polarization of ≈30 μC cm^2^^[^ ^42^ ^]^ and a rapid switching time of ≈70 ns. This combination matches or surpasses the best‐performing HZO films reported to date. These results demonstrate that careful control of the oxidation atmosphere in mixed Ar/O_2_ during PLD can be used to overcome the conventional trade‐off between high polarization and fast switching.

## 3 Conclusion

This work highlights the critical role of redox conditions in the preparation of epitaxial ferroelectric Hf_0.5_Zr_0.5_O_2_ films by PLD on their ferroelectric switching dynamics. We demonstrate that precise tuning of oxygen pressure directly impacts domain nucleation, domain wall mobility, and phase stabilization, likely related to oxygen vacancy concentration variation. Meanwhile, the introduction of additional inert argon gas reduces the switching time, indicating that high‐energy plasma effects intrinsic to PLD impact film crystallinity and defect landscape uniformity, and thus the analyzed functional properties. The combined optimization of these parameters achieves a remarkable synergy, enabling both fast switching kinetics and high polarization, which are two performance metrics that have historically been in competition. The study reveals that oxygen vacancies and/or defects do not favor fast switching. Overall, this study provides a comprehensive framework for overcoming conventional performance trade‐offs in HfO_2_‐based ferroelectrics, advancing their applicability in high‐speed, reliable memory and neuromorphic systems.

## 4 Experimental Section

Hf_0.5_Zr_0.5_O_2_ films, with thicknesses ranging from 7.7 to 10.2 nm, were grown on STO substrates buffered with a LSMO electrode of ≈25 nm. The ferroelectric Hf_0.5_Zr_0.5_O_2_ film and the LSMO electrode were grown in a single process by PLD using a KrF excimer laser. LSMO electrodes were deposited under an oxygen pressure of 0.1 mbar at a substrate temperature of 700 °C. The Hf_0.5_Zr_0.5_O_2_ films were deposited under an Ar/O_2_ atmosphere at 800 °C. Three series of films were grown by varying the oxygen pressure (*P*_O2_) with fixed Ar pressure (*P*_Ar_) values of 0, 0.05, and 0.1 mbar. The sketch in Supporting Information Figure S11 summarizes the *P*_Ar_/*P*_O2_ values in these series. The films were cooled under 0.2 mbar of oxygen immediately after growth. Circular platinum top electrodes, with a diameter of 20 μm and a thickness of 20 nm, were deposited by DC magnetron sputtering through stencil masks for electrical characterization. The electrode size of the samples grown under 0.002, 0.005, 0.01, 0.02, and 0.05 mbar oxygen pressure and 0.1 mbar argon pressure is 400, 383, 349, 430, and 283 μm^2^, respectively.

The crystal structure was characterized by XRD with Cu K*α* radiation using a Bruker D8‐Discover diffractometer equipped with a point detector. XPS measurements were carried out in an ultrahigh vacuum chamber (≈1 × 10^−8^ mbar) using a Physical Electronics Genesis spectrometer equipped with a monochromatic Al K*α* X‐ray source (hν = 1486.6 eV). The photoelectrons were collected at a take‐off angle of 90° relative to the surface normal. Samples were exposed to air during transfer from the PLD chamber to the XPS chamber. Energy calibration was performed by referencing the adventitious C 1*s* peak at 284.8 eV. The Hf 4*f* core‐level spectra were fitted using XPSPEAK software, applying a Shirley‐type background and mixed Gaussian–Lorentzian line shapes. Ferroelectric and switching spectroscopy characterization was performed using a TFAnalyzer3000 platform (Aixacct GmbH), grounding the LSMO bottom electrode and biasing one of the top Pt electrodes. The pre‐cycling was performed by applying a bipolar triangular pulse of 4.5 V at a frequency of 1 kHz before switching to spectroscopy characterizations. Supporting Information Figure S12 shows the pulse sequence used for switching spectroscopy, and details on the parameters used are summarized in Supporting Information Table S2. The details of voltage and time parameters for write and read pulses used for switching spectroscopy of Hf_0.5_Zr_0.5_O_2_ capacitors are shown in (Table S1, Supporting Information). Under illumination experiments were collected by exposing the device under measurement to blue–violet light of *λ* = 405 nm, *E*_photon_ = 3.06 eV, Power density ≈12.2 W cm^2^. For Rayleigh analysis, small‐signal polarization–voltage (*P*–*V*) hysteresis loops were measured under varying AC voltage amplitudes using the TFAnalyzer3000 system. The measurements were performed at a fixed frequency of 1 kHz with voltage amplitudes kept below the coercive voltage. The dielectric permittivity was extracted from the slope of the polarization loops, and the field dependence was analyzed to determine the Rayleigh parameters.

## Supporting Information

Supporting Information is available from the Wiley Online Library or from the author.

## Conflict of Interest

The authors declare no conflict of interest.

## Supporting information

Supplementary Material

## Data Availability

The data that support the findings of this study are available from the corresponding author upon reasonable request.

## References

[1] S. H. Park , H. J. Lee , M. H. Park , J. Kim , H. W. Jang , J. Phys. D Appl. Phys. 2024, 57, 253002.

[2] L. W. Martin , A. M. Rappe , Nat. Rev. Mater. 2016, 2, 16087.

[3] T. Mikolajick , S. Slesazeck , H. Mulaosmanovic , M. H. Park , S. Fichtner , P. D. Lomenzo , M. Hoffmann , U. Schroeder , J. Appl. Phys. 2021, 129, 100901.

[4] J. P. B. Silva , R. Alcala , U. E. Avci , N. Barrett , L. Bégon‐Lours , M. Borg , S. Byun , S.‐C. Chang , S.‐W. Cheong , D.‐H. Choe , J. Coignus , V. Deshpande , A. Dimoulas , C. Dubourdieu , I. Fina , H. Funakubo , L. Grenouillet , A. Gruverman , J. Heo , M. Hoffmann , H. A. Hsain , F.‐T. Huang , C. S. Hwang , J. Íñiguez , J. L. Jones , I. V. Karpov , A. Kersch , T. Kwon , S. Lancaster , et al., APL Mater. 2023, 11, 089201.

[5] S. González‐Casal , I. Fina , F. Sánchez , J. Fontcuberta , ACS Appl. Electron. Mater. 2019, 1, 1937.

[6] T. Mikolajick , M. H. Park , L. Begon‐Lours , S. Slesazeck , Adv. Mater. 2023, 35, 2206042. 10.1002/adma.202206042 36017895

[7] U. Schroeder , M. H. Park , T. Mikolajick , C. S. Hwang , Nat. Rev. Mater. 2022, 7, 653.

[8] F. Ali , T. Ali , D. Lehninger , A. Sünbül , A. Viegas , R. Sachdeva , A. Abbas , M. Czernohorsky , K. Seidel , Adv. Funct. Mater. 2022, 32, 2201737.

[9] F. Ali , A. Abbas , G. Wu , M. Daaim , A. Akhtar , K. Kim , B. Yang , Small 2022, 18, 2200133. 10.1002/smll.202200133 35445535

[10] K. D. Kim , M. H. Park , H. J. Kim , Y. J. Kim , T. Moon , Y. H. Lee , S. D. Hyun , T. Gwon , C. S. Hwang , J. Mater. Chem. C Mater. 2016, 4, 6864.

[11] M. Materano , C. Richter , T. Mikolajick , U. Schroeder , J. Vac. Sci. Technol. A 2020, 38, 022402.

[12] H. A. Hsain , Y. Lee , M. Materano , T. Mittmann , A. Payne , T. Mikolajick , U. Schroeder , G. N. Parsons , J. L. Jones , J. Vac. Sci. Technol. A 2022, 40, 010803.

[13] T. Mittmann , M. Materano , P. D. Lomenzo , M. H. Park , I. Stolichnov , M. Cavalieri , C. Zhou , C. Chung , J. L. Jones , T. Szyjka , M. Müller , A. Kersch , T. Mikolajick , U. Schroeder , Adv. Mater. Interfaces 2019, 6, 1900042.

[14] K. Lee , K. Park , H.‐J. Lee , M. S. Song , K. C. Lee , J. Namkung , J. H. Lee , J. Park , S. C. Chae , Sci. Rep. 2021, 11, 6290. 33737670 10.1038/s41598-021-85773-7 PMC7973512

[15] Y.‐C. Li , X.‐X. Li , Z.‐Y. Huang , X.‐N. Zhu , D. W. Zhang , H.‐L. Lu , IEEE Electron Device Lett. 2024, 45, 829.

[16] M. Materano , T. Mittmann , P. D. Lomenzo , C. Zhou , J. L. Jones , M. Falkowski , A. Kersch , T. Mikolajick , U. Schroeder , ACS Appl. Electron. Mater. 2020, 2, 3618.

[17] D. H. Lee , Y. Lee , K. Yang , J. Y. Park , S. H. Kim , P. R. S. Reddy , M. Materano , H. Mulaosmanovic , T. Mikolajick , J. L. Jones , U. Schroeder , M. H. Park , Appl. Phys. Rev. 2021, 8, 021312.

[18] Y.‐C. Li , T. Huang , X.‐X. Li , X.‐N. Zhu , D. W. Zhang , H.‐L. Lu , Nano Lett. 2024, 24, 6585. 38785400 10.1021/acs.nanolett.4c00263

[19] Y. Li , J. Li , R. Liang , R. Zhao , B. Xiong , H. Liu , H. Tian , Y. Yang , T.‐L. Ren , Appl. Phys. Lett. 2019, 114, 142902.

[20] S.‐N. Choi , S.‐E. Moon , S.‐M. Yoon , Ceram. Int. 2019, 45, 22642.

[21] M. Si , X. Lyu , P. R. Shrestha , X. Sun , H. Wang , K. P. Cheung , P. D. Ye , Appl. Phys. Lett. 2019, 115, 072107. 10.1063/1.5098786 PMC11194797 38915734

[22] Y. Sawabe , T. Saraya , T. Hiramoto , C.‐J. Su , V. P.‐H. Hu , M. Kobayashi , Appl. Phys. Lett. 2022, 121, 082903.

[23] Y. Peng , Z. Wang , W. Xiao , Y. Ma , F. Liu , X. Deng , X. Yu , Y. Liu , G. Han , Y. Hao , Ceram. Int. 2022, 48, 28489.

[24] P. Hao , S. Zheng , B. Zeng , T. Yu , Z. Yang , L. Liao , Q. Peng , Q. Yang , Y. Zhou , M. Liao , Adv. Funct. Mater. 2023, 33, 2301746.

[25] M. C. Sulzbach , H. Tan , S. Estandía , J. Gàzquez , F. Sánchez , I. Fina , J. Fontcuberta , ACS Appl. Electron. Mater. 2021, 3, 3657.

[26] B. Prasad , V. Thakare , A. Kalitsov , Z. Zhang , B. Terris , R. Ramesh , Adv. Electron. Mater. 2021, 7, 2001074.

[27] J. Lyu , T. Song , I. Fina , F. Sánchez , Nanoscale 2020, 12, 11280. 32420576 10.1039/d0nr02204g

[28] J. Lyu , I. Fina , R. Solanas , J. Fontcuberta , F. Sánchez , ACS Appl. Electron. Mater. 2019, 1, 220.

[29] H. Tan , S. Estandía , F. Sánchez , I. Fina , ACS Appl. Electron. Mater. 2023, 5, 6630. 10.1021/acsaelm.2c01186 PMC9979785 36873260

[30] T. Song , F. Sánchez , I. Fina , APL Mater. 2022, 10, 031108.

[31] J. Lyu , I. Fina , F. Sánchez , Appl. Phys. Lett. 2020, 117, 072901.

[32] T. Mittmann , M. Michailow , P. D. Lomenzo , J. Gärtner , M. Falkowski , A. Kersch , T. Mikolajick , U. Schroeder , Nanoscale 2021, 13, 912. 33367444 10.1039/d0nr07699f

[33] Y. H. Lee , H. J. Kim , T. Moon , K. Do Kim , S. D. Hyun , H. W. Park , Y. Bin Lee , M. H. Park , C. S. Hwang , Nanotechnology 2017, 28, 305703. 28562366 10.1088/1361-6528/aa7624

[34] A. Pal , V. K. Narasimhan , S. Weeks , K. Littau , D. Pramanik , T. Chiang , Appl. Phys. Lett. 2017, 110, 022903.

[35] J. Gonzalo , J. Siegel , A. Perea , D. Puerto , V. Resta , M. Galvan‐Sosa , C. N. Afonso , Phys. Rev. B 2007, 76, 035435.

[36] M. Mirjolet , F. Sánchez , J. Fontcuberta , Adv. Funct. Mater. 2019, 29, 1808432.

[37] B. Shin , M. J. Aziz , Phys. Rev. B 2007, 76, 085431.

[38] P. R. Willmott , R. Herger , C. M. Schlepütz , D. Martoccia , B. D. Patterson , Phys. Rev. Lett. 2006, 96, 176102. 16712314 10.1103/PhysRevLett.96.176102

[39] V. Trtík , A. Pérez , J. Navarro , C. Ferrater , F. Sánchez , M. Varela , Appl. Phys. A Mater. Sci. Process. 1999, 69, S815.

[40] J. Gonzalo , C. N. Afonso , J. Perrière , Appl. Phys. Lett. 1995, 67, 1325.

[41] S. Wicklein , A. Sambri , S. Amoruso , X. Wang , R. Bruzzese , A. Koehl , R. Dittmann , Appl. Phys. Lett. 2012, 101, 131601.

[42] T. Song , R. Solanas , M. Qian , I. Fina , F. Sánchez , J. Mater. Chem. C Mater. 2022, 10, 1084.

[43] F. Ali , T. Song , I. Fina , F. Sánchez , Appl. Phys. Lett. 2024, 124, 142903.

[44] A. Chernikova , M. Kozodaev , A. Markeev , D. Negrov , M. Spiridonov , S. Zarubin , O. Bak , P. Buragohain , H. Lu , E. Suvorova , A. Gruverman , A. Zenkevich , ACS Appl. Mater. Interfaces 2016, 8, 7232. 26931409 10.1021/acsami.5b11653

[45] W. Hamouda , A. Pancotti , C. Lubin , L. Tortech , C. Richter , T. Mikolajick , U. Schroeder , N. Barrett , J. Appl. Phys. 2020, 127, 064105.

[46] X. Long , H. Tan , S. Estandía , J. Gazquez , F. Sánchez , I. Fina , J. Fontcuberta , APL Mater. 2022, 10, 031114.

[47] M. Kumar , H. Seo , Adv. Mater. 2022, 34, 2106881. 10.1002/adma.202106881 34725878

[48] H. Tan , A. Quintana , N. Dix , S. Estandía , J. Sort , F. Sánchez , I. Fina , Nano Energy 2024, 123, 109384.

[49] T. Song , P. Koutsogiannis , C. Magén , J. A. Pardo , F. Sánchez , I. Fina , Adv. Electron. Mater. 2024, 10, 2300509.

[50] I. Fina , L. Fàbrega , E. Langenberg , X. Martí , F. Sánchez , M. Varela , J. Fontcuberta , J. Appl. Phys. 2011, 109, 074105.

[51] X. Lyu , J. Zhou , F. Ali , I. Fina , F. Sánchez , Small 2025, 21, e06334. 40772370 10.1002/smll.202506334 PMC12462559

[52] J. Y. Jo , H. S. Han , J.‐G. Yoon , T. K. Song , S.‐H. Kim , T. W. Noh , Phys. Rev. Lett. 2007, 99, 267602. 18233604 10.1103/PhysRevLett.99.267602

[53] T. H. Kim , S. H. Baek , S. M. Yang , Y. S. Kim , B. C. Jeon , D. Lee , J.‐S. Chung , C. B. Eom , J.‐G. Yoon , T. W. Noh , Appl. Phys. Lett. 2011, 99, 012905.

[54] H. Shin , V. Gaddam , Y. Goh , Y. Jeong , G. Kim , Y. Qin , S. Jeon , Appl. Phys. Lett. 2023, 122, 022901.

[55] R. Cao , Q. Liu , M. Liu , B. Song , D. Shang , Y. Yang , Q. Luo , S. Wu , Y. Li , Y. Wang , H. Lv , IEEE Electron Device Lett. 2019, 40, 1744.

[56] Y. Goh , J. Hwang , M. Kim , Y. Lee , M. Jung , S. Jeon , ACS Appl. Mater. Interfaces 2021, 13, 59422. 34855347 10.1021/acsami.1c14952

[57] H. P. Bonzel , T. E. Fischer , Surf. Sci. 1975, 51, 213.

[58] Y. Q. Zhan , I. Bergenti , L. E. Hueso , V. Dediu , M. P. de Jong , Z. S. Li , Phys. Rev. B 2007, 76, 045406.

[59] D. Lee , B. C. Jeon , A. Yoon , Y. J. Shin , M. H. Lee , T. K. Song , S. D. Bu , M. Kim , J. Chung , J. Yoon , T. W. Noh , Adv. Mater. 2014, 26, 5005. 24847984 10.1002/adma.201400654

[60] P. Maksymovych , N. Balke , S. Jesse , M. Huijben , R. Ramesh , A. P. Baddorf , S. V. Kalinin , J. Mater. Sci. 2009, 44, 5095.

[61] V. P. Afanasjev , A. A. Petrov , I. P. Pronin , E. A. Tarakanov , E. J. Kaptelov , J. Graul , J. Phys.: Condens. Matter 2001, 13, 8755.

[62] H. Tan , G. Castro , J. Lyu , P. Loza‐Alvarez , F. Sánchez , J. Fontcuberta , I. Fina , Mater. Horiz. 2022, 9, 2345. 35968715 10.1039/d2mh00644h

[63] B. C. Jeon , D. Lee , M. H. Lee , S. M. Yang , S. C. Chae , T. K. Song , S. D. Bu , J. Chung , J. Yoon , T. W. Noh , Adv. Mater. 2013, 25, 5643. 23897638 10.1002/adma.201301601

[64] W. J. Merz , Phys. Rev. 1954, 95, 690.

[65] M. G. Farahani , A. Quintana , T. Song , R. Kumar , A. Rubano , F. Ali , F. Sánchez , I. Fina , ACS Appl. Mater. Interfaces 2025, 17, 3570. 39742420 10.1021/acsami.4c15867

[66] D. A. Hall , Ferroelectrics 1999, 223, 319.

[67] Y. Guan , D. Zhou , J. Xu , X. Liu , F. Cao , X. Dong , J. Müller , T. Schenk , U. Schroeder , Phys. Status Solidi ‐ RRL 2015, 9, 589.

[68] D. A. Hall , P. J. Stevenson , Ferroelectrics 1999, 228, 139.

[69] D. A. Hall , J. Mater. Sci. 2001, 36, 4575.

[70] Y. Song , J. Yu , Z. Wang , K. Xu , Y. Liu , C. Wang , K. Chen , Q. Sun , D. Wei Zhang , L. Chen , IEEE Electron Device Lett. 2025, 46, 12.

[71] J. Liu , B. Zeng , Q. Yang , Z. Yang , T. Yu , C. Ju , S. Zheng , Q. Peng , Y. Zhou , Q. Yang , M. Liao , J. Mater. Sci. Technol. 2026, 241, 311.

[72] H. Liu , T. Lu , Y. Li , Z. Ju , R. Zhao , J. Li , M. Shao , H. Zhang , R. Liang , X. R. Wang , R. Guo , J. Chen , Y. Yang , T. Ren , Adv. Sci. 2020, 7, 2001266. 10.1002/advs.202001266 PMC7539221 33042746

[73] C. Alessandri , P. Pandey , A. Abusleme , A. Seabaugh , IEEE Electron Device Lett. 2018, 39, 1780.

[74] B. Buyantogtokh , V. Gaddam , S. Jeon , J. Appl. Phys. 2021, 129, 244106.

[75] T. Chiang , J. J. Plombon , M. K. Lenox , I. Mercer , P. Debashis , M. D. C. S. Trolier‐McKinstry , J.‐P. Maria , J. F. Ihlefeld , I. A. Young , J. T. Heron 2025, https://arxiv.org/abs/2507.12353.

