# Supplementary material for: Fast Switching and High Polarization in Ferroelectric Hf0.5Zr0.5O2 Films
Source: Small Sci. 2026 Jan 15;6(2):e202500465. doi: 10.1002/smsc.202500465 (PMC12910630; doi:10.1002/smsc.202500465)
Supplement: Supplementary file 1 — Supplementary Material [file SMSC-6-e202500465-s001.pdf]

Fast Switching and High Polarization in Ferroelectric $Hf_{0.5}Zr_{0.5}O_2$ Films

Faizan Ali,[1,*] Tingfeng Song,[2,3] , Florencio Sánchez,[1,*] Ignasi Fina[1,*]

[1]Institut de Ciència de Materials de Barcelona (ICMAB-CSIC), Campus UAB, Bellaterra 08193, Spain

[2]Department of Applied Physics, The Hong Kong Polytechnic University, Hong Kong, China

[3]Joint Research Center of Microelectronics, The Hong Kong Polytechnic University, Hong Kong, China

Corresponding authors: I. Fina (ifina@icmab.es), F. Sánchez (fsanchez@icmab.es), F. Ali (faizanali@icmab.es)

Supporting information

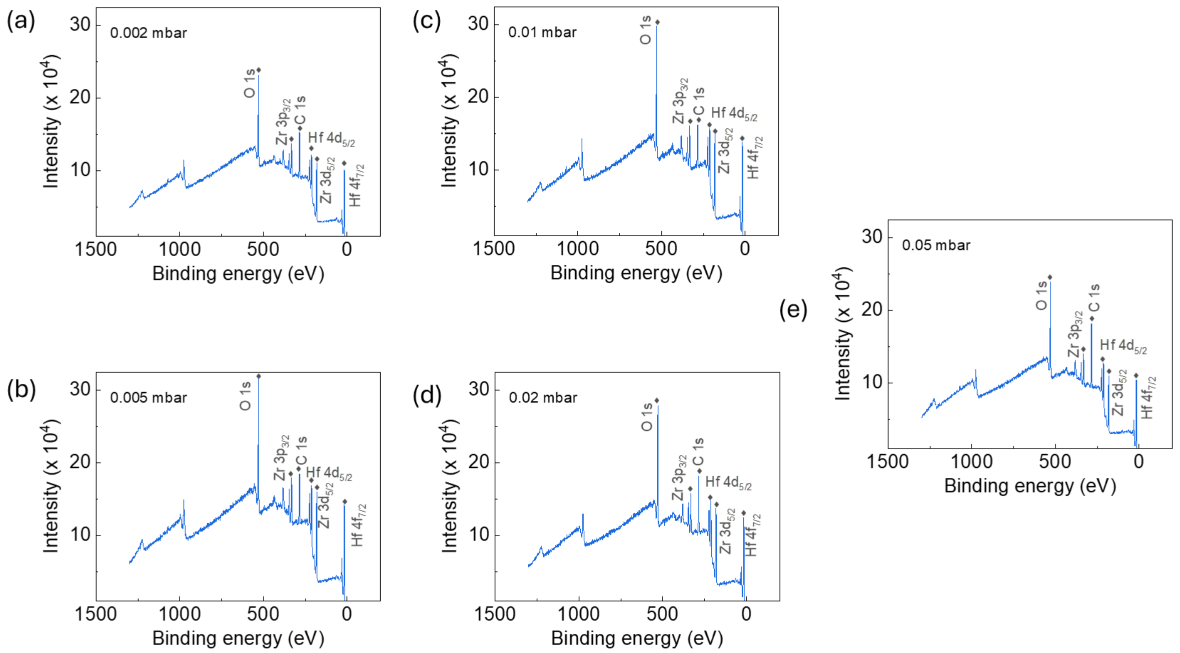

Figure S1. Survey XPS spectra of $Hf_{0.5}Zr_{0.5}O_2$ films deposited at 0.1 mbar Ar pressure and oxygen pressure of (a) 0.002 mbar, (b) 0.005 mbar, (c) 0.01 mbar, (d) 0.02 mbar, and (e) 0.05 mbar.

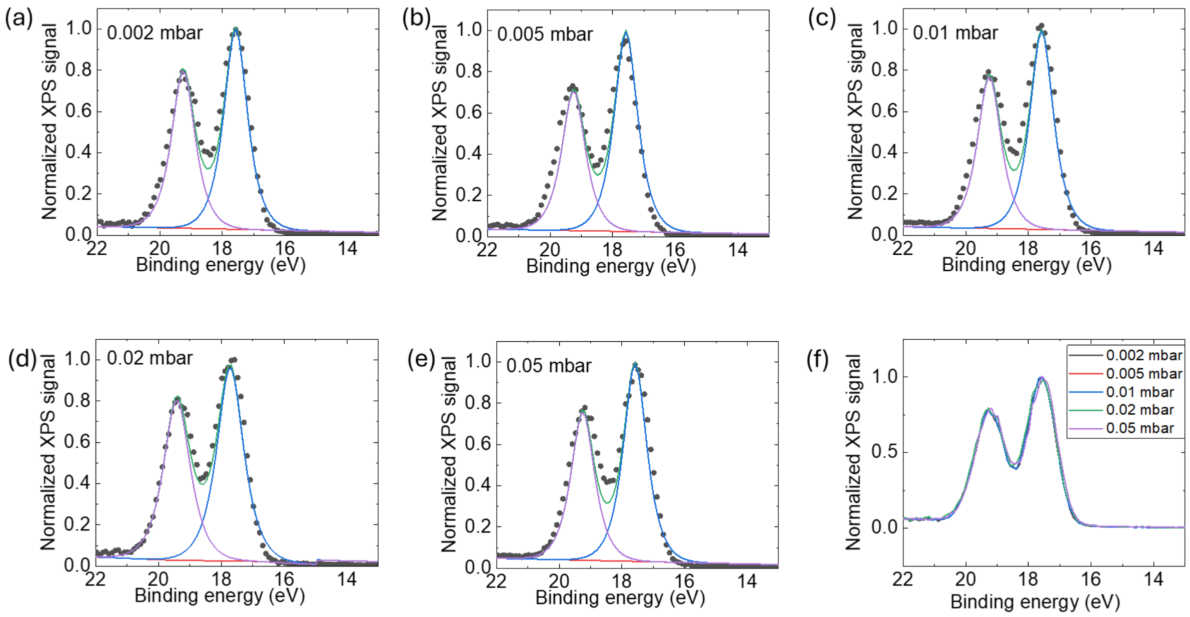

Figure S2. XPS Hf 4f core spectra of $Hf_{0.5}Zr_{0.5}O_2$ films deposited at 0.1 mbar Ar pressure and oxygen pressure of (a) 0.002 mbar, (b) 0.005 mbar, (c) 0.01 mbar, (d) 0.02 mbar, and (e) 0.05 mbar. The spectra exhibit the characteristic Hf^{4+} spin-orbit doublet ($4f_{7/2}$ = 17.58 eV; $4f_{5/2} \approx$ 19.2 eV), with no detectable sub-oxide contributions, consistent with stoichiometric HfO_2. The black circles represent the experimental data. The blue and purple curves correspond to the fitted $4f_{7/2}$ and $4f_{5/2}$ components, respectively; the red line represents the Shirley-type background, and the green line denotes the total fitted envelope. (f) Normalized XPS Hf 4f spectra of $Hf_{0.5}Zr_{0.5}O_2$ films deposited at argon pressure of 0.1 mbar and varying oxygen partial pressure (0.002-0.05 mbar). The close overlap of the normalized curves indicates that the Hf oxidation state remains unchanged with varying oxygen pressure.

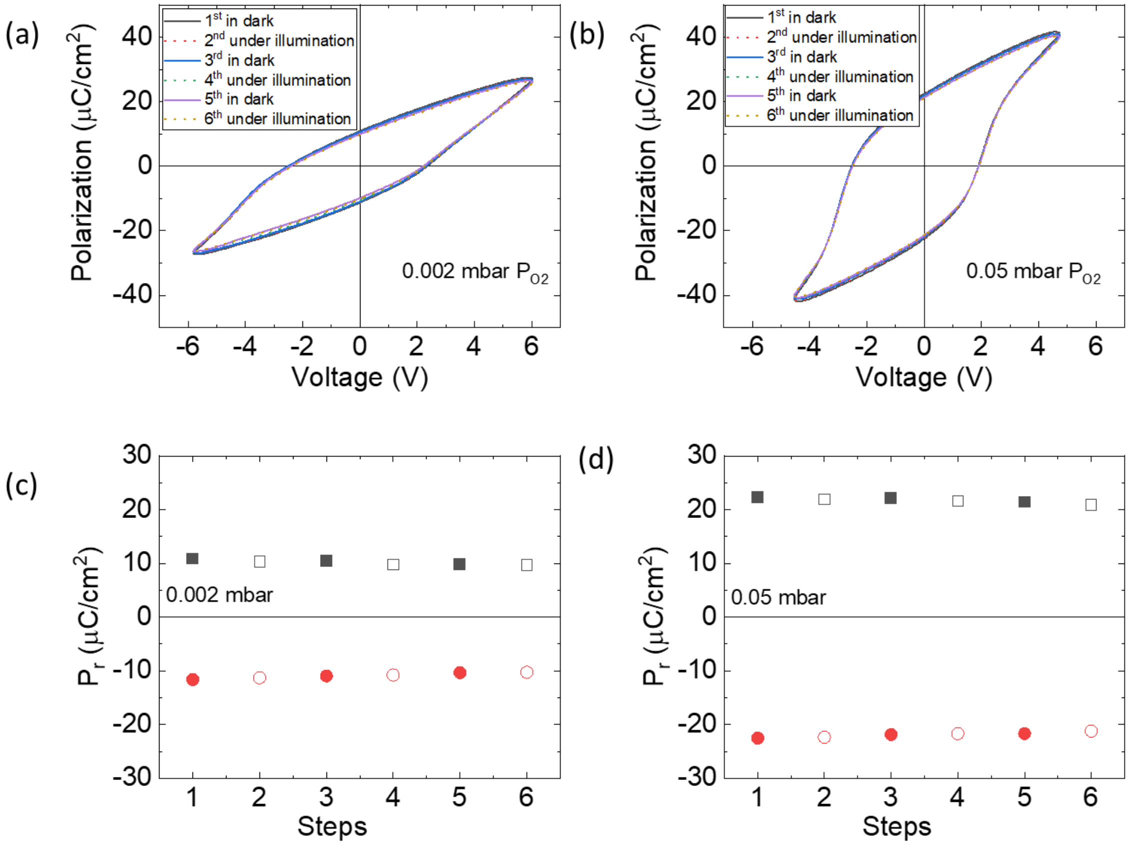

Figure S3. Ferroelectric polarization-voltage (P-V) hysteresis loops and corresponding remanent polarization (P_r^+ and P_r^-) evolution of $Hf_{0.5}Zr_{0.5}O_2$ films measured with and without light illumination for films deposited at (a, c) 0.002 mbar and (b, d) 0.05 mbar oxygen pressure (P_{Ar} = 0.1 mbar). Panels (a) and (b) show six consecutive P-V loops recorded alternating in the dark (solid lines) and under illumination (dashed lines) conditions, while panels (c) and (d) display the extracted P_r^+ and P_r^- values as a function of measurement step (1-6). Filled symbols correspond to measurements in the dark, and open symbols represent data obtained under illumination. The overlapping loops and nearly constant P_r values confirm that light exposure does not measurably influence the ferroelectric switching behavior or polarization stability in either film.

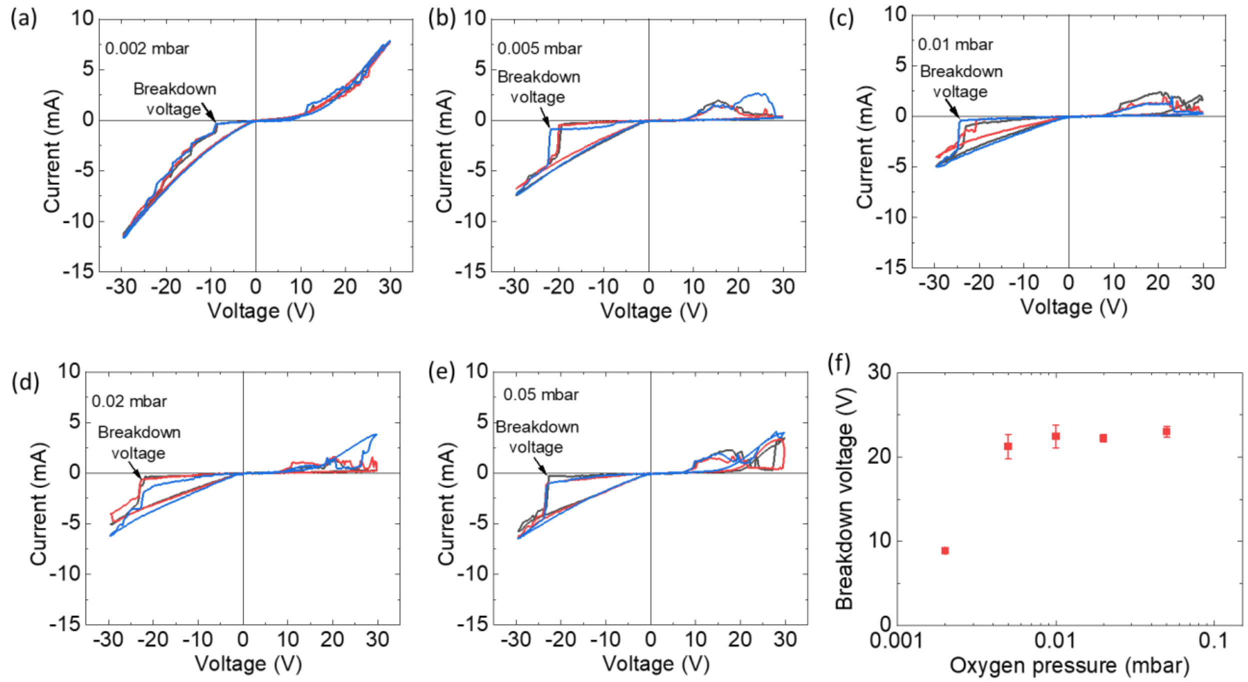

Figure S4. I-V curves collected at high voltage of $Hf_{0.5}Zr_{0.5}O_2$ films deposited at 0.1 mbar Ar pressure and oxygen pressure of (a) 0.002 mbar, (b) 0.005 mbar, (c) 0.01 mbar, (d) 0.02 mbar, and (e) 0.05 mbar. (f) Breakdown voltage of $Hf_{0.5}Zr_{0.5}O_2$ films as a function of oxygen partial pressure during deposition at a fixed argon pressure of 0.1 mbar. The error bars in (f) represent the standard deviation calculated from three independent measurements for each condition.

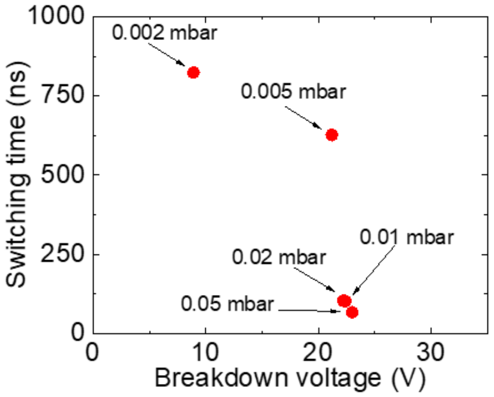

Figure S5. The relationship between breakdown voltage and switching time of $Hf_{0.5}Zr_{0.5}O_2$ films deposited at 0.1 mbar Ar pressure and O_2 pressure ranging from 0.002 to 0.05 mbar.

Leakage correction procedure

To accurately extract the switched polarization (ΔP) of the $Hf_{0.5}Zr_{0.5}O_2$ capacitors, we applied a fitting procedure that accounts for the residual leakage current contribution, which is not completely removed by the conventional Positive-Up Negative-Down (PUND) method. The experimental ΔP was fitted using the Nucleation-Limited Switching (NLS) model, modified to include a logarithmic leakage term:

$$\Delta P = 2P_r \int_{-\infty}^{+\infty} \left[1 - e^{-(\frac{t}{\tau})^n}\right] F(\log \tau) \times d(\log \tau) + Q. \log(\tau_w) \quad (1)$$

where n is the effective dimension (fixed at n =2), τ is the characteristic switching time, and F(log τ) is the Lorentzian distribution for the logarithm of switching time. The Lorentzian distribution can be expressed as:

$$F(log\tau) = \frac{A}{\pi}\left[\frac{w}{(log\tau - logt_1)^2 + w^2}\right] \quad (2)$$

where A is a normalization constant, w is the full width at half maxima (FWHM), and log t_1 is the central value of the Lorentzian distribution for the logarithm of switching time. The additional $Q. \log(\tau_w)$ term represents the phenomenological residual leakage current contribution, where Q is a constant.

The corrected switched polarization was obtained by subtracting the fitted $Q. \log(\tau_w)$ term from the raw ΔP data. This term captures the residual logarithmic increase in polarization at longer writing times due to coexisting electronic and ionic leakage mechanisms, which cause hysteretic conductivity (resistive switching) in hafnia-based films. After subtraction, the corrected ΔP curves saturate as expected, confirming that the remaining signal corresponds to true ferroelectric switching. Figure S2 compares the experimental ΔP data before and after leakage subtraction for

an $Hf_{0.5}Zr_{0.5}O_2$ sample deposited at 0.05 mbar P_{Ar} and 0.05 mbar P_{O2}, demonstrating the effectiveness of this correction.

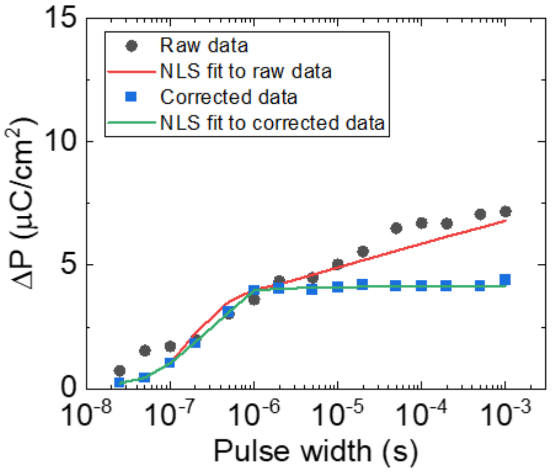

Figure S6. Comparison of experimental switched polarization (ΔP) versus writing pulse width for an $Hf_{0.5}Zr_{0.5}O_2$ sample deposited at 0.05 mbar P_{Ar} and 0.05 mbar P_{O2}, shown before and after subtraction of the fitted residual leakage contribution.

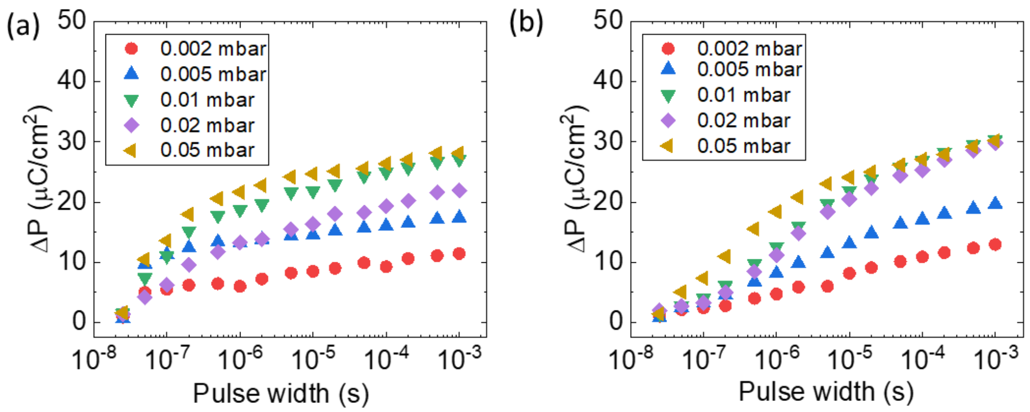

Figure S7. Switched polarization (ΔP) as a function of pulse width for $Hf_{0.5}Zr_{0.5}O_2$ films deposited at constant Ar pressure (0.1 mbar) and varying oxygen pressures (0–0.05 mbar): (a) under +4 V and (b) under -4 V writing bias. These measurements provide raw switching data before leakage current correction.

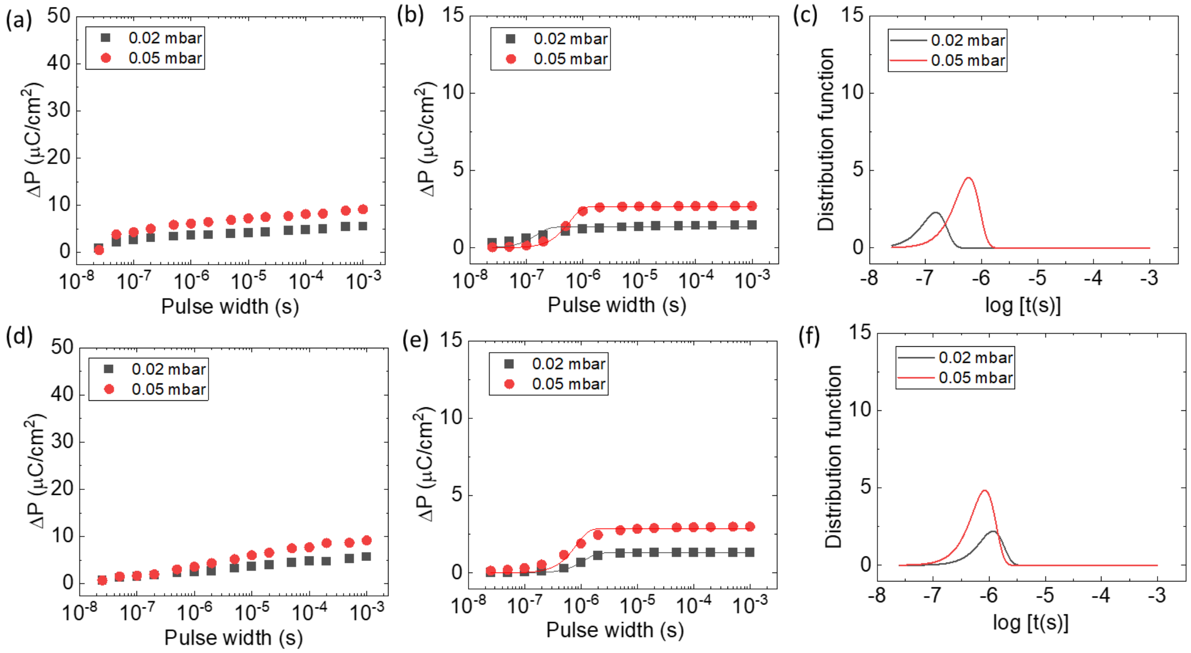

Figure S8. Switching dynamics of $Hf_{0.5}Zr_{0.5}O_2$ films deposited at constant Ar pressure (0 mbar) and varying oxygen pressures (0.02 and 0.05 mbar): (a) Raw switched polarization (ΔP) vs. writing pulse width under +4 V (before leakage correction), (b) leakage-corrected ΔP vs. writing pulse width under +4 V with NLS model fitting, (c) corresponding Lorentzian distribution functions of switching time under +4 V, (d) raw ΔP vs. writing pulse width under –4 V, (e) leakage-corrected ΔP vs. writing pulse width under –4 V with NLS model fitting, and (f) Lorentzian distribution functions of log [t(s)] under –4 V.

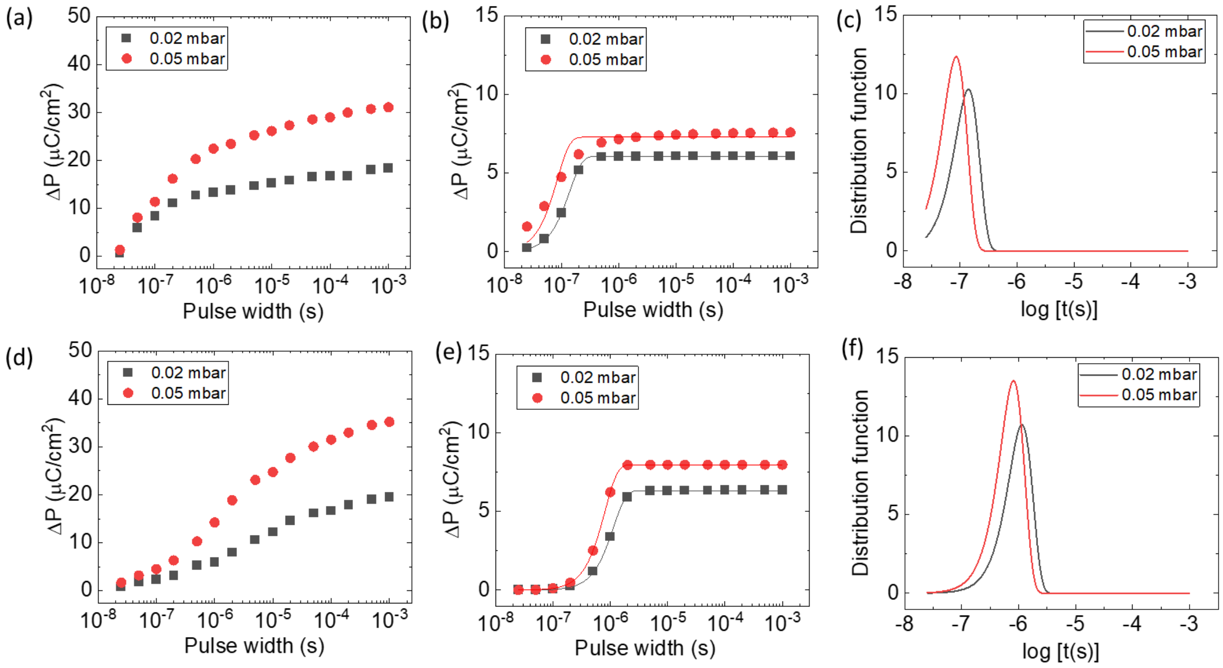

Figure S9. Switching dynamics of $Hf_{0.5}Zr_{0.5}O_2$ films deposited at constant Ar pressure (0.05 mbar) and varying oxygen pressures (0.02 and 0.05 mbar): (a) raw switched polarization (ΔP) vs. writing pulse width under +4 V (before leakage correction), (b) leakage-corrected ΔP vs. writing pulse width under +4 V with NLS model fitting, (c) corresponding Lorentzian distribution functions of switching time under +4 V, (d) raw ΔP vs. writing pulse width under –4 V, (e) leakage-corrected ΔP vs. writing pulse width under –4 V with NLS model fitting, and (f) Lorentzian distribution functions of log[t(s)] under –4 V.

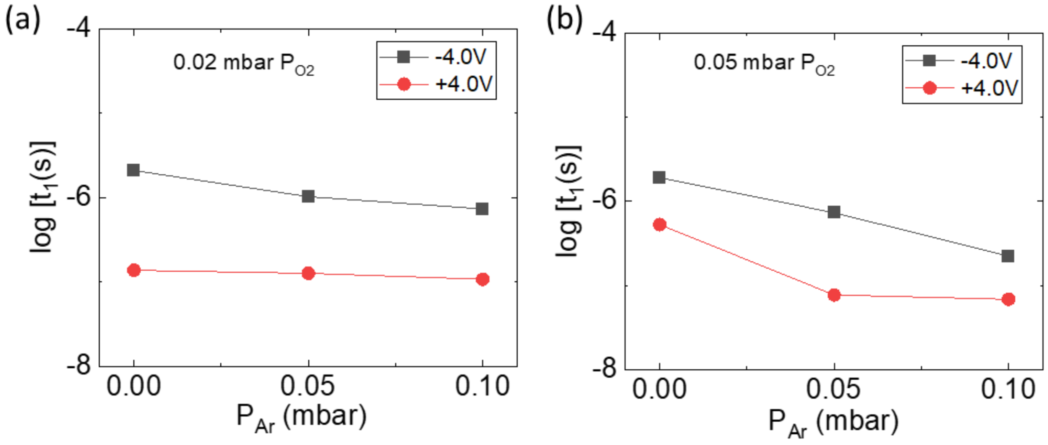

Figure S10. (a) log [t_1(s)] as a function of Ar pressure (0, 0.05, and 0.1 mbar) at a fixed oxygen pressure of 0.02 mbar, for both positive and negative writing polarities and (b) log [t_1(s)] as a function of Ar pressure at a fixed oxygen pressure of 0.05 mbar, for both positive and negative writing polarities.

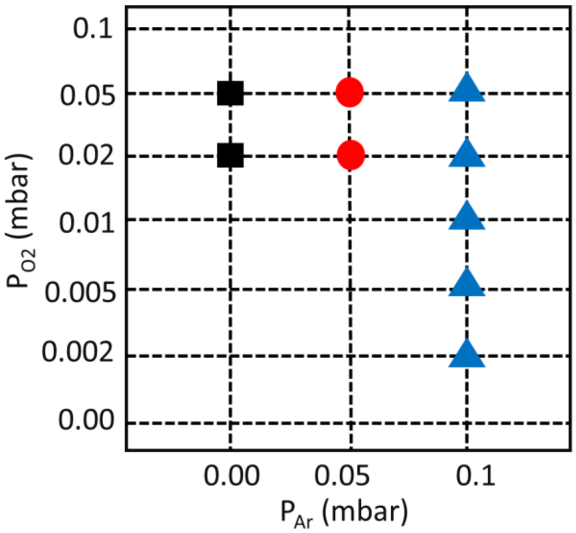

Figure S11. Summary of the three series of $Hf_{0.5}Zr_{0.5}O_2$ films deposited at fixed Ar partial pressures: black squares represent films grown at P_{Ar} = 0 mbar, red circles at P_{Ar} = 0.05 mbar, and blue triangles at P_{Ar} = 0.1 mbar. Each series includes samples with varying oxygen partial pressures (P_{O2}).

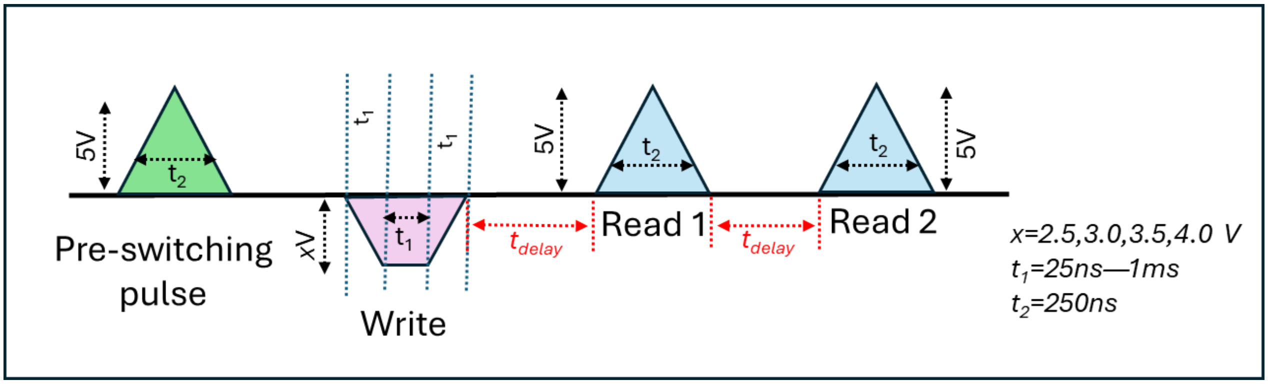

Figure S12. Schematic illustration of the pulse train sequence used for switching spectroscopy measurements. The sequence includes preset, write, and read pulses designed to probe the time-dependent polarization response of $Hf_{0.5}Zr_{0.5}O_2$ capacitors under varying voltages.

Table S1. Comparison of remanent polarization and switching time for $Hf_{1-x}Zr_xO_2$-based ferroelectric thin films reported in previous studies and in this work. The results highlight the broad range of switching kinetics achieved through different compositional and structural engineering strategies.

**Composition**	**P_r (μC/cm^2)**	**Switching time**	**Reference**
Tri layers $Hf_{0.3}Zr_{0.7}O_2$, $Hf_{0.5}Zr_{0.5}O_2$, and $Hf_{0.3}Zr_{0.7}O_2$	17	0.78 ns	[1]
composition-graded $Hf_{0.7}Zr_{0.3}O_2$, $Hf_{0.5}Zr_{0.5}O_2$, and $Hf_{0.3}Zr_{0.7}O_2$.	13	330 ns	[2]
$Hf_{0.5}Zr_{0.5}O_2$	19.6	100 ns	[3]
$Hf_{0.5}Zr_{0.5}O_2$	15	10 ns	[4]
$Hf_{0.5}Zr_{0.5}O_2$	16	890 ns	[5]
$Hf_{0.5}Zr_{0.5}O_2$	20	236 ns	[6]
$Hf_{0.5}Zr_{0.5}O_2$	24.5	66.5 ns	[7]
$Hf_{0.5}Zr_{0.5}O_2$	20	0.22 ns	[8]
$Hf_{0.5}Zr_{0.5}O_2$	27.8	67 ns	**This work**

Table S2. Voltage amplitudes and timing parameters used for the write and read pulses in switching spectroscopy measurements of $Hf_{0.5}Zr_{0.5}O_2$ capacitors.

**Parameter**	**Write pulse**	**Read pulse**
**Voltage**	Varied from ±4.0 V to ±2.5 V, with a step of 0.5 V.	±5.0 V
**Pulse width**	Varied from 25 ns to 1 ms	triangular pulses are used.
**Rise/decay time**	Same as writing pulse width	250μs
**Delay time**	1 s	1 s

References

[1] Y. Song, J. Yu, Z. Wang, K. Xu, Y. Liu, C. Wang, K. Chen, Q. Sun, D. Wei Zhang, L. Chen, *IEEE Electron Device Letters* 2025, *46*, 12.

[2] P. Hao, S. Zheng, B. Zeng, T. Yu, Z. Yang, L. Liao, Q. Peng, Q. Yang, Y. Zhou, M. Liao, *Adv Funct Mater* 2023, *33*, 2301746.

[3] J. Liu, B. Zeng, Q. Yang, Z. Yang, T. Yu, C. Ju, S. Zheng, Q. Peng, Y. Zhou, Q. Yang, M. Liao, *J Mater Sci Technol* 2026, *241*, 311.

[4] M. Si, X. Lyu, P. R. Shrestha, X. Sun, H. Wang, K. P. Cheung, P. D. Ye, *Appl Phys Lett* 2019, *115*, 072107.

[5] H. Liu, T. Lu, Y. Li, Z. Ju, R. Zhao, J. Li, M. Shao, H. Zhang, R. Liang, X. R. Wang, R. Guo, J. Chen, Y. Yang, T. Ren, *Advanced Science* 2020, *7*.

[6] C. Alessandri, P. Pandey, A. Abusleme, A. Seabaugh, *IEEE Electron Device Letters* 2018, *39*, 1780.

[7] B. Buyantogtokh, V. Gaddam, S. Jeon, *J Appl Phys* 2021, *129*.

[8] T. Chiang, J. J. Plombon, M. K. Lenox, I. Mercer, P. Debashis, M. DC, S. Trolier-McKinstry, J.-P. Maria, J. F. Ihlefeld, I. A. Young, J. T. Heron, 2025.
